# Neuropathic-like symptoms and central sensitization related signs and symptoms negatively affect the functional performance of patients with knee osteoarthritis – a cross-sectional study

**DOI:** 10.1016/j.ocarto.2023.100358

**Published:** 2023-04-05

**Authors:** Enrico Seixas Goldoni, Juliana Valentim Bittencourt, Lanucia Ranhol do Espirito Santo, Eduardo Branco de Sousa, José Leonardo Rocha de Faria, Dângelo José de Andrade Alexandre, Leandro Alberto Calazans Nogueira

**Affiliations:** a Rehabilitation Science Postgraduate Programme at Augusto Motta University Centre (UNISUAM), Rio de Janeiro, Brazil; b Physiotherapy Department at Federal Institute of Rio de Janeiro (IFRJ), Rio de Janeiro, Brazil; c Physiotherapy Department at Jamil Haddad National Institute of Traumatology and Orthopedics (INTO), Rio de Janeiro, Brazil

**Keywords:** Osteoarthritis, Knee, Neuropathic pain, Central sensitization, Functionality

## Abstract

**Objective:**

This study aimed to compare the functional performance among participants with a neuropathic-like symptoms (NS) and central sensitization related signs and symptoms (CS), and their knee osteoarthritis (OA) counterparts.

**Methods:**

A cross-sectional observational study was conducted with 125 participants with knee OA (94 females, mean age 63.1 ​± ​7.4 years). Participants completed a self-reported questionnaire with personal and clinical features and musculoskeletal pain characteristics, including NS (PainDETECT), CS (Central Sensitization Inventory, CSI), and conditioned pain modulation. Self-reported functional disability (Western Ontario and McMaster Universities Osteoarthritis Index, WOMAC) and functional mobility (Timed Up and Go, TUG) were compared among patients with NS, CS, and their knee OA counterparts using the one-way analysis of variance (ANOVA).

**Results:**

Thirty-three (26.4%) participants had NS and CS, eighteen (14.4%) had NS, twenty-seven (21.6%) participants had CS, and 47 (37.6%) had knee OA with no NS or CS. A one-way ANOVA revealed greater functional limitation in the group with NS and CS (mean ​= ​67.5 ​± ​12.0) or NS (mean ​= ​56.7 ​± ​17.5) than the group without these symptoms (mean ​= ​32.0 ​± ​20.7) with a statistical significance difference [F(3, 121) ​= ​29.434, p ​< ​0.001] in the WOMAC Total score. The group with NS and CS (mean ​= ​19.2 ​± ​7.4) or NS (mean ​= ​16.3 ​± ​6.3) had slower velocity than the group without these symptoms (mean ​= ​11.6 ​± ​3.5) with a statistical significance difference [F(3,121) ​= ​10.045, p ​< ​0.001] in the TUG test.

**Conclusion:**

Participants with knee osteoarthritis and NS or CS pain phenotype have greater functional limitations than their counterparts.

## 1 Introduction

Knee Osteoarthritis (OA) is one of the leading causes of disability in the elderly [1]. Globally, almost 10% of the population over 60 years is affected by OA; 80% of this population presents movement restrictions, and 25% have functional limitations that compromise the performance of daily activities [2,3]. Clinically, knee OA is associated with pain, stiffness, deformity, and loss of functional capacity [4,5]. Most OA patients have functional limitations, such as morning stiffness, reduced joint mobility, crackles, and muscle atrophy [6]. Therefore, investigating aspects of the functionality of patients with knee OA is relevant.

OA pain has nociceptive and neuropathic mechanisms at the local and central levels [7]. OA arises from a complex biological process involving structural changes in the local tissues (i.e., cartilage, bone, ligaments, capsule, synovial membrane, meniscus, and periarticular muscles) [8,9]. Longer nociceptive input may also lead to changes in central pain processing and increase the likelihood of developing neuropathic pain [10]. Neuropathic-like symptoms (NS) tend to be more frequent in OA patients with a diffuse and medial posterior pattern than in medial anterior pain [11]. Recent studies indicate that patients with OA and preoperative neuropathic pain have higher pain levels after knee arthroplasty [12,13]. Furthermore, patients with neuropathic pain have a more significant functional impairment, multimodal hyperalgesia, and greater pain sensitivity [14]. Therefore, measuring the impact of NS on the functionality of patients with knee OA can assist therapeutic management of this population.

Patients with knee OA have also presented central sensitization related signs and symptoms (CS) [15]. CS corresponds to impaired functioning of descending inhibitory mechanisms projected by the brain and hyperactivation of pain facilitating pathways [16]. Impaired conditioned pain modulation is an important feature of the CS [17]. Patients with knee OA and altered central pain modulation before surgery have an increased risk of unfavorable outcomes after knee arthroplasty [[18], [19], [20]]. Likewise, the Central Sensitization Inventory (CSI) is a screening tool developed to identify patients with CS [21]. Patients with knee OA and high CSI scores had greater pain intensity three months after total knee arthroscopy compared to those with low CSI scores [22]. Chronic pain before surgery can change the somatosensory system, impaired pain modulation and increase the risk of maintaining it after surgery [13] Thus, identifying the CS in patients with knee OA could provide insights regarding the need for additional treatment strategies.

OA requires a broad assessment, including screening elements affecting functional performance. Prior researchers investigated the isolated influence of neuropathic symptoms or central pain on pain intensity, persistent pain after surgery or the predictive ability of these pain features. However, the same patient with knee OA may present local findings indicating neuropathic pain and central pain component simultaneously. Therefore, this study aimed to compare the functional performance among participants with NS, CS, and their knee OA counterparts. We hypothesized that participants with knee OA plus NS or CS have impaired functional performance more significant when compared to their counterparts. Further, participants with NS and CS would present even unfavorable functional performance since these conditions are not mutually exclusive.

## 2 Methods

### 2.1 Study design and ethical considerations

This is a cross-sectional study design reported following the Strengthening the Reporting of Observational studies in Epidemiology (STROBE) requirements [23]. This study was approved by the Research Ethics Committee of Augusto Motta University Center (UNISUAM) (number 48067621.0.0000.5235) and of Jamil Haddad National Institute of Traumatology and Orthopaedics (INTO) (number 48067621.0.3001.5273) in accordance with the Helsinki Declaration for research in humans. All patients met the eligibility criteria signed the informed consent form before the study procedures.

### 2.2 Eligibility criteria

Participants with knee OA who met the American College of Rheumatology (ACR) clinical criteria and self-reported knee pain for more than six months were included. The clinical criteria of the ACR require at least three of the following six clinical findings, morning knee stiffness lasting less or equal to 30 ​min, age over 50 years, palpable/audible crepitus in active movement, tenderness to palpation of bone margins joints, bone enlargement on physical examination, and absence of palpable local heat [24].

Participants were recruited in the waiting rooms of two outpatient Physical Therapy departments. These participants were referred or on the waiting list for knee replacement surgery. Participants with a self-reported history of lower limb prosthesis; and self-report cognitive impairment, inflammatory rheumatic disorder, osteosynthesis, neoplasms and metabolic diseases (Paget), severe structural injuries in the acute phase, severe comorbidities such as uncompensated heart disease (coronary, ischemic, cerebrovascular, peripheral arterial, rheumatic heart/valvular disease, and congenital heart disease) and also decompensated respiratory (chronic obstructive pulmonary disease, and poorly controlled asthma, interstitial lung diseases with cystic fibrosis complications with recurrent infections) and/or neuromuscular (muscular dystrophies); and individuals using assistive walking devices (such as canes, crutches, and walkers) were excluded.

### 2.3 Procedures

Consecutive participants with knee OA from two outpatient Physical Therapy departments (UNISUAM and INTO) were screened. Data collection on clinical history, sociodemographic (age, sex, weight, height, education level, and income), and musculoskeletal pain characteristics (pain intensity and pain duration) were performed using a standard questionnaire. NS were measured by the painDETECT questionnaire and CS were assessed by Central Sensitization Inventory (CSI). Conditioned Pain Modulation (CPM) was investigated using the Cold Pressor Test. Participants were instructed to immerse one hand in a bucket with temperature-controlled cold water (1 ​°C – 4 ​°C) monitored by a thermometer (5130 model, Incoterm) for up to 1 ​min. Pressure pain threshold was performed before and after 1 ​min of the cold pressor test, using a digital pressure algometer (model SP Tech, MedOR, Medtech, Santa Catarina, Brazil). The distal part of the dorsal forearm and tibialis anterior muscle, which had not been immersed in water, were chosen to be evaluated. Only participants with the inefficiency of the CPM in both locations (the anterior tibialis muscle and the distal part of the dorsal forearm) were classified as impaired pain modulation. Functional performance considered two aspects: functional disability and functional mobility. Functional disability was measured using the Western Ontario and McMaster Universities Osteoarthritis Index (WOMAC) and functional mobility was assessed by the Timed Up and Go test (TUG). All participants were evaluated by the same examiner (E.S.G.), a physical therapist with 13 years of clinical experience in musculoskeletal rehabilitation.

### 2.4 Exposures

#### 2.4.1 Neuropathic-like symptoms

The painDETECT is a self-administered questionnaire developed to differentiate NS from nociceptive pain components in participants with chronic low back pain. PainDETECT has been validated for many neuropathic pain conditions in mixed pain conditions (neuropathic and nociceptive characteristics), such as rheumatoid arthritis, OA, cancer, and lumbar spondylolisthesis [25]. PainDETECT questionnaire was adapted cross-culturally to the Brazilian context [25]. The questionnaire has a sensitivity of 85% and a specificity of 80% for identifying NS in participants with low back pain [26]. The cutoff points for the original questionnaire indicate that at scores ≤12, a neuropathic component is unlikely, while at scores ≥19, a neuropathic component is likely [26]. PainDETECT encompasses four domains as follows: intensity of the pain (three questions), pain course pattern (four graphs), areas of pain and the presence of radiating pain (body chart drawing), and sensory descriptor items of pain (seven questions). The final score is calculated by nine-item represented in the last three domains (pain course pattern, radiating pain, and gradation of pain). A final score between −1 and 38 can be achieved by summing up the scores given in each domain.

### 2.5 CS-related signs and symptoms

The Central Sensitization Inventory (CSI) identified patients whose presentation symptoms may be related to central sensitization. CSI is an instrument developed to identify CS-related signs and symptoms [21]. Part A assesses 25 health-related symptoms commonly observed in patients with central sensitivity syndrome and is scored on a 5-point Likert scale from 0 (never) to 4 (always), with a total of 100 points. Higher scores represent an increase in the severity of symptoms. Part B is not scored and encompasses ten previous diagnoses of an individual, including seven central sensitivity syndromes and three disorders related to central sensitization syndrome. The optimal cutoff point was established at 40/100 in patients with central sensitivity syndrome [27,28]. The Brazilian version of the CSI demonstrated strong psychometric properties [29].

### 2.6 Main outcome measures

#### 2.6.1 Functional disability

The Western Ontario and McMaster Universities Osteoarthritis Index (WOMAC) is a multidimensional questionnaire used to assess pain (5 items), stiffness (2 items), and physical function (17 items) in participants with hip or knee OA. WOMAC has already been translated and validated for different languages, including Brazilian Portuguese [30]. This questionnaire focuses on symptoms and physical function in participants with knee and hip problems [31]. The WOMAC questionnaire was administered through face-to-face interviews. All items have the same weight, and each item has a score ranging from 0 (none) to 4 (extreme). Each domain can be scored separately, and the total questionnaire score ranges from 0 to 96, where 0 is the best and 96 is the worst score. The Portuguese-Brazilian WOMAC showed good overall quality for assessing symptoms and knee function [32].

### 2.7 Functional mobility

The Timed Up and Go test (TUG) was used to assess functional mobility, and the test execution time was recorded using a stopwatch. The TUG was developed as a basic test for functional mobility and consists of measuring speed during various maneuvers provided, which include standing, walking, turning, and sitting [33]. The TUG is a reliable, fast, and easy-to-perform test to measure dynamic balance [34]. The participant must be seated in a chair with lateral arm support (with a height of 46 ​cm from the seat to the floor and a height of 65 ​cm from the armrest to the floor) and is instructed to stand up without leaning on the sides of the chair, walk 3 ​m, turn 180° and return to the starting point to sit again [[35], [36], [37], [38]] [[35], [36], [37], [38]] [[35], [36], [37], [38]]. Participants who fail to complete the test in less than 13.5 ​s are at greater risk of falling [39]. The time and speed were used to report the functional mobility performance.

### 2.8 Research funding

This study was financed in part by the Fundação Carlos Chagas Filho de Amparo à Pesquisa do Estado do Rio de Janeiro [Grant number: E−26/211.104/2021] and Coordenação de Aperfeiçoamento de Pessoal de Nível Superior - Brasil [Finance Code 001; Grant number: 88881.708719/2022–01, grant number: 88887.708718/2022–00, and grant number 88887.466981/2019–00].

### 2.9 Sample size calculation

The sample size was estimated based on findings of Polat et al. (2017) [40], who compared physical disability measured by the WOMAC between participants with knee OA with and without a NS measured by the painDETECT questionnaire. The effect size of 0.4 was obtained from the WOMAC total score of participants classified as having probable NS and participants with unlikely neuropathic pain. Considering a significance level of 0.05 and a power of 95%, a total sample of 112 participants was estimated using the one-way analysis of variance (ANOVA), including four groups. Sample calculation was performed using the G∗Power software version 3.1.9 (Heinrich-Heine Universität, Düsseldorf, Germany).

### 2.10 Statistical analysis

Descriptive analysis of sociodemographic and clinical data was performed. Continuous variables were presented as mean and standard deviation (SD). Categorical variables were presented as absolute values and proportions (%). The comparison of functional performance among patients with NS (painDETECT scores ≥19), CS (CSI scores ≥40), and their knee OA counterparts was performed using the one-way ANOVA. Dunnet's post hoc was used after ANOVA, if necessary. The fourth group comprised participants with neuropathic-like symptoms plus central sensitization-related signs and symptoms. Then, participants with a painDETECT questionnaire score of ≥19 points and a CSI score of ≥40 points formed the fourth group. We added a fourth group to the comparison formed by participants with NS and CS since these conditions are not mutually exclusive. The analysis was performed using JASP software (version 0.16.1 for Mac open-source, free license). The graph was made using the GraphPad Prism software (Version X8. Oa, San Diego, CA, USA). A significance level of less than 5% (P ​< ​0.05) was considered for all analyses.

## 3 Results

**Fig. 1:**
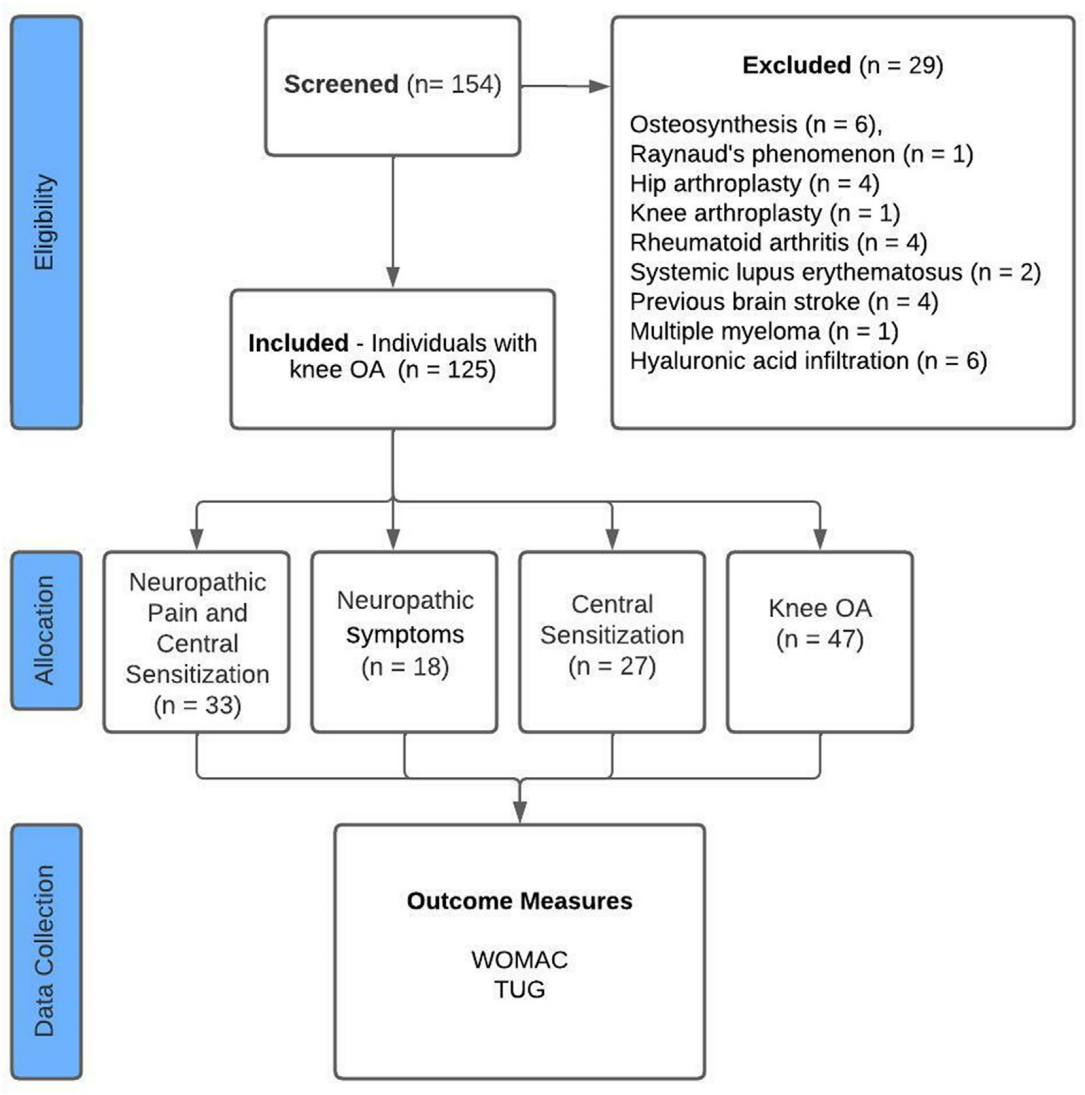
Flowchart of the study participants.

A total of 154 participants were screening and 29 were excluded due to the osteosynthesis (n ​= ​6), Raynaud's phenomenon (n ​= ​1), hip arthroplasty (n ​= ​4), knee arthroplasty (n ​= ​1), rheumatoid arthritis (n ​= ​4), systemic lupus erythematosus (n ​= ​2), previous brain stroke (n ​= ​4), multiple myeloma (n ​= ​1) and hyaluronic acid infiltration (n ​= ​6). Fig. 1 shows the flowchart of the participants included in the study. One hundred twenty-five participants with knee OA fulfilled the eligibility criteria, being 94 (75%) females with a mean age of 63.1 ​± ​7.4 years, and 51 (41%) reported practice physical activities regularly.

Thirty-three (26.4%) participants were classified as NS plus CS, eighteen (14.4%) as NS, twenty-seven (21.6%) as CS, and 47 (37.6%) as knee OA purely (with no NS or CS). Regarding to the pain features, NS plus CS and the NS groups demonstrated higher pain intensity than participants with knee OA purely. Still, pain duration was similar among the groups. NS plus CS and CS groups demonstrated greater impairment of conditioned pain modulation compared to participants with knee OA purely. Table 1 presents the comparison between participants with NS plus CS, NS, CS or knee OA purely, respectively.

**Table 1:** Comparison of the participants' characteristics, pain features and functional performance among participants with with knee OA with or without neuropathic-like symptoms and CS-related signs and symptoms (n ​= ​125).

Characteristic	NS plus CS group (n ​= ​33, 26.4%)	NS group (n ​= ​18, 14.4%)	CS group (n ​= ​27, 21.6%)	Knee OA (n ​= ​47, 37.6%)	p-value
Sex, n (%), female	32 (97.0)	13 (72.2)	24 (88.9)	25 (53.2)^a^^,^^b^	<0.001
Age, mean (SD)	62.7 (6.1)	65.4 (6.1)	62.2 (8.9)	63.5 (7.8)	0.418
Weight (kg), mean (SD)	84.8 (20.3)	76.8 (12.8)	80.4 (17.7)	81.5 (13)	0.412
Height (m), mean (SD)	1.63 (0.1)	1.65 (0.1)	1.61 (0.1)	1.65 (0.1)	0.268
Body Mass Index (kg/m^2^), mean (SD)	31.9 (7.0)	28.2 (3.1)	31.2 (5.7)	30.2 (5.3)	0.171
Physical Exercise (Yes), n (%)	13 (39.4)	8 (44.4)	9 (33.3)	21 (44.7)	0.791
Risk of falling, yes, n (%)	23 (69.7)	12 (66.7)	13 (48.1)	12 (25.6)^a^	<0.001
Pain duration (months), mean (SD)	111.0 (79.9)	100.3 (73.9)	106.8 (125.6)	90.1 (80.9)	0.750
Pain intensity, mean (SD)	7.8 (2.3)	7.4 (2.2)	5.7 (3.4)	4.2 (3.3)^a^^,^^b^^,^^c^	<0.001
Final score PainDETECT, mean (SD)	24.4 (4.1)	23.1 (3.7)	12.7 (4.3)	9.2 (4.7)^a^^,^^b^^,^^c^	<0.001
CSI, mean (SD)	56.9 (11.6)	30.2 (7.3)	51.4 (7.6)	23.3 (8.2)^a^^,^^b^^,^^c^	<0.001
Cold Pressor Test, yes, n (%)	23 (69.7)	7 (38.9)	20 (74.1)	11 (23.4)^a^^,^^b^^,^^c^	<0.001
WOMAC					
Pain assessment, mean (SD)	12.6 (2.3)	9.6 (3.9)	9.8 (3.6)	6.4 (3.9)^a^^,^^b^^,^^c^	<0.001
Stiffness assessment, mean (SD)	5.7 (1.5)	4.7 (2.7)	3.8 (2.1)	3.0 (2.3)^a^^,^^b^	<0.001
Physical function assessment, mean (SD)	49.2 (9.7)	42.4 (13.9)	33.3 (13.0)	22.7 (15.8)^a^^,^^b^^,^^c^	<0.001

Note: Data are presented as mean (SD) for continuous variables and frequency counts (%) for categorical variables. The comparison of functional performance among the groups was performed using the one-way ANOVA.

a Represents a significant difference between the knee osteoarthritis group and neuropathic-like symptoms plus central sensitization-related signs and symptoms group.

b Represents a significant difference between the knee osteoarthritis group and neuropathic-like symptoms group.

c Represents a significant difference between knee osteoarthritis group and central sensitization-related signs and symptoms group. Abbreviations: NS, neuropathic-like symptoms; CS, central sensitization-related signs and symptoms; CSI, Central Sensitization Inventory; WOMAC, Western Ontario and McMaster Universities Osteoarthritis Index.

### 3.1 Comparison of functional disability and functional mobility

**Fig. 2:**
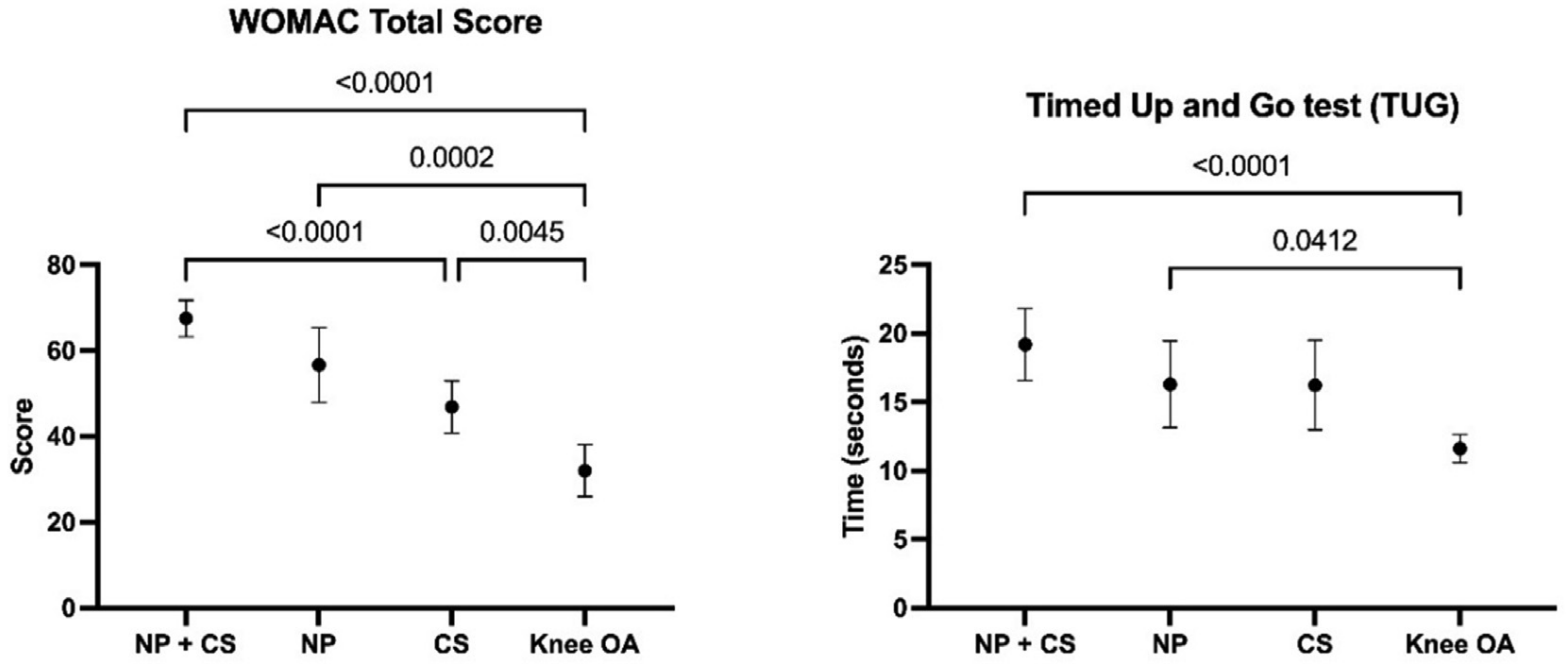
Comparison of the Western Ontario and McMaster Universities Osteoarthritis Index domains and The Timed Up and Go test between participants with knee osteoarthritis with neuropathic-like symptoms, central sensitization-related signs and symptoms, neuropathic-like symptoms plus central sensitization-related signs and symptoms, and without neuropathic-like symptoms or central sensitization-related signs and symptoms (n ​= ​125).

A one-way ANOVA revealed greater functional limitation in the group with NS plus CS (mean ​= ​67.5, SD ​= ​12.0) or NS (mean ​= ​56.7, SD ​= ​17.5) than the participants with knee OA without NS or CS (mean ​= ​32.0, SD ​= ​20.7) with a statistical significance difference [F(3, 121) ​= ​29.434, p ​< ​0.001, η^2^ ​= ​0.42] in the WOMAC total score. The group with NS plus CS (mean ​= ​19.2, SD ​= ​7.4) or NS (mean ​= ​16.3, SD ​= ​6.3) had slower velocity than the participants with knee OA without NS or CS (mean ​= ​11.6, SD ​= ​3.5) with a statistical significance difference [F(3,121) ​= ​10.045, p ​< ​0.001, η^2^ ​= ​0.20] in the TUG test. Participants with CS without NS had significantly higher self-reported functional limitation measured by the WOMAC but a non-significant difference in the speed during the TUG test than those with knee OA without NS or CS (Dunnet's test, p ​= ​0.005 and p ​= ​0.051, respectively) (see Fig. 2).

## 4 Discussion

The current study aimed to compare the functional performance among participants with a NS, CS, NS plus CS, and their knee OA counterparts. Our findings confirm the hypothesis that the presence of NS or CS negatively affect the functional performance of participants with knee OA. Participants with CS had a deficient physical performance perception and a trend to slower functional mobility than those with knee OA solely.

NS are associated with poor outcomes in knee OA such as increased pain severity, disability [[40], [41], [42]] and greater functional deficit [11,43]. We found a higher functional limitation in patients with knee OA and NS measured by patient's perception and objectively. Similarly, a previous study showed that patients with knee OA who score highly on the painDETECT presented increased pain intensity and greater functional impairment than the remaining cohort [14]. Moreover, patients with knee OA and NS had longer symptom duration, lower physical function and quality of life [42]. Therefore, the presence of NS negatively impacts the functional performance of patients with knee OA.

In the current study, participants with knee OA and CS had greater self-perception functional impairment, confirming prior research showing that CSI scores correlated strongly with the WOMAC pain scores and moderately with the WOMAC function in patients undergoing total knee arthroplasty for knee OA [44]. In agreement, many patients with knee OA continued to experience CS after knee arthroplasty [45]. Moreover, these patients demonstrated a lower rate of minimal clinically important difference for the WOMAC score after total knee arthroplasty than patients without CS [46]. Notably, the participants with CS presented a self-perception of physical limitation but a slight reduction in functional mobility measured by TUG. Thus, CS affects more functional self-perception than objective functional mobility.

Participants with local findings indicating neuropathic pain and central pain component presented a particular pain phenotype. These participants exhibited greater functional limitation than participants with knee OA purely, but similar functional capacity than participants with NS. Likewise, pain intensity and risk of falling were higher in participants with NS plus CS than participants with knee OA purely and identical to participants with NS. Accordingly, the presence of neuropathic component may play a crucial role to the severity of the functional capacity, pain intensity, and risk of falling in patients with knee OA. On the other hand, the impairment of conditioned pain modulation was greater in the two groups with CS compared to participants with knee OA without these symptoms. Most of the participants classified with CS clinically had impaired conditioned pain modulation despite this impairment in the other groups. Indeed, the CS phenotype is more closely related to psychological features than conditioned pain modulation [47]. Ultimately, participants with CS share clinical similarities with participants with NS, but particular limitations in the pain modulation and self-perceived functional performance, indicate a specific phenotype of patients with knee OA.

Identifying distinct pain phenotypes in patients with knee OA is endorsed to treat these patients adequately [9]. The phenotype with neuropathic plus central pain component share similarities with patients with NS, except for the conditioned pain modulation. Measuring the factors that affect the functionality in patients waiting for knee replacement may contribute to assertive decision-making. In this sense, the presence of NS or CS leads to an unfavoured clinical outcomes in patients with knee OA. Future studies should consider a particular pain phenotype of knee OA with neuropathic-like symptoms and central sensitization to evaluate the potential impact of this pain phenotype on the clinical outcomes in other settings or countries to confirm our initial findings.

We recognize the strengths and limitations of the current study. This is the first study to examine the functional performance in patients with knee OA, NS plus CS concomitantly. Besides, we compared the functional performance subjectively and objectively among different knee OA pain phenotypes. Knowing the pain features which impact a patient's functionality assists the surgeons in choosing the appropriate therapy for knee OA candidates for joint replacement. On the other hand, the absence of diagnostic imaging represents a limitation. Nonetheless, previous studies showed that structural lesions are not associated with clinical outcomes [48,49], and there is no correlation between radiological changes and response to treatment [48]. Moreover, we used the clinical diagnostic criteria following the American College of Rheumatology [50]. Another limitation is using self-reported questionnaires to detect neuropathic and central pain components. Neurological examination and quantitative sensory tests should be implemented in future studies to confirm our findings.

In summary, participants with knee osteoarthritis and NS or CS pain phenotype have greater functional limitations than their counterparts.

## Author contributions

Conceptualisation, ESG., JVB., and L.A.C.N.; Methodology, ESG., JVB., LRES., and L.A.C.N.; Investigation, ESG., JVB., LRES., and L.A.C.N.; Writing – Original Draft, ESG., JVB., EBS., JLRF., DJAA., and L.A.C.N.; Writing – Review & Editing, ESG., JVB., EBS., JLRF., DJAA., and L.A.C.N.

## Research funding

This study was financed in part by the Fundação Carlos Chagas Filho de Amparo à Pesquisa do Estado do Rio de Janeiro (FAPERJ) [Grant number: E-26/211.104/2021] and Coordenação de Aperfeiçoamento de Pessoal de Nível Superior - Brasil (CAPES) [Finance Code 001; Grant number: 88881.708719/2022-01, grant number: 88887.708718/2022-00, and grant number 88887.466981/2019-00].

## Conflict of interest

Authors state no conflict of interest.
